# Iron Selenide Particles for High-Performance Supercapacitors

**DOI:** 10.3390/ma16155309

**Published:** 2023-07-28

**Authors:** Davide Scarpa, Claudia Cirillo, Eleonora Ponticorvo, Carla Cirillo, Carmine Attanasio, Mariagrazia Iuliano, Maria Sarno

**Affiliations:** 1 Department of Physics “E.R. Caianiello”, University of Salerno, Via Giovanni Paolo II, 132, 84084 Fisciano, Italy; clcirillo@unisa.it (C.C.); eponticorvo@unisa.it (E.P.); cattanasio@unisa.it (C.A.); maiuliano@unisa.it (M.I.); 2 NANO_MATES Research Centre, University of Salerno, Via Giovanni Paolo II, 132, 84084 Fisciano, Italy; 3 CNR-SPIN, c/o University of Salerno, Via Giovanni Paolo II, 132, 84084 Fisciano, Italy; carla.cirillo@spin.cnr.it

**Keywords:** metal selenide electrode, FeSe, high capacitance, energy density

## Abstract

Nowadays, iron (II) selenide (FeSe), which has been widely studied for years to unveil the high-temperature superconductivity in iron-based superconductors, is drawing increasing attention in the electrical energy storage (EES) field as a supercapacitor electrode because of its many advantages. In this study, very small FeSe particles were synthesized via a simple, low-cost, easily scalable, and reproducible solvothermal method. The FeSe particles were characterized using cyclic voltammetry (CV), galvanostatic charge/discharge (GCD) measurements, and electrochemical impedance spectroscopy (EIS), revealing enhanced electrochemical properties: a high capacitance of 280 F/g at 0.5 A/g, a rather high energy density of 39 Wh/kg and a corresponding power density of 306 W/kg at 0.5 A/g, an extremely high cycling stability (capacitance retention of 92% after 30,000 cycles at 1 A/g), and a rather low equivalent series resistance (R_ESR_) of ~2 Ω.

## 1. Introduction

In the last few decades, energy storage technology has attained fundamental relevance as a consequence of the increased global demand for electrical energy, allowing surplus energy storage from intermittent renewable sources into the electrical grid. To this extent, energy storage has played a key role in increasing the efficiency and cost-effectiveness of the electrical grid by integrating the sustainable energy supply [1].

In the field of electrochemical energy storage, supercapacitor-based technology, filling the gap between batteries/fuel cells and traditional capacitors, is becoming very promising, owing to some intrinsic advantages: lower costs, very fast charge/discharge rates, remarkable power densities (more than 10 kW/kg), superior long cycle lives (more than 10^6^ cycles), wide-ranging temperatures, and increased environmental safety [2]. Supercapacitors can be classified as (i) electrical double-layer capacitors (EDLCs) [3] and (ii) redox capacitors [4,5].

Regarding EDLCs, the energy storage principle is based on the formation of a double layer of ions from the electrolyte solution close to the electrode surfaces, known as the Helmholtz electrical double layer. Such an energy storage mechanism guarantees high power densities, owing to the fast kinetics of the adsorption and desorption of ions, but lower capacitances. On the other hand, in redox capacitors, the energy storage mechanism is based on redox reactions carried out at the interfaces between the electrodes and electrolyte, which also occur in the bulk of the electrode and enable this type of supercapacitor to have a higher capacitance than EDLCs. However, because of the slow kinetics of redox reactions, redox capacitors have reduced cycle stability and lower power densities than EDLCs; therefore, selecting high-performance Faradaic materials is an excellent strategy to overcome the limits of these devices.

Among redox materials, ruthenium oxide (RuO_2_) is one of the most commonly employed materials at the electrode, Refs. [6,7] even though its industrial-scale application has been hindered by its high price, amounting to nearly 90% of the production cost of a whole device [8]. To reduce such a high cost, several metal oxides (such as SnO_2_, MnO_2_, NiO, VOx, TiO_2_, MoO_3_, WO_3_, Fe_3_O_4,_ and CaO) have been proposed recently in combination with RuO_2_ or as substitutions [9].

On the other hand, transition metal chalcogenides with the formula of TMX (with TM = transition metal and X = S, Se, or Te) have been explored as electrode materials in the field of renewable energy to enhance energy storage capabilities due to their large numbers of redox sites, remarkable electrical conductivity, peculiar crystal structures, and outstanding physicochemical properties compared to the transition metal oxide [10,11,12,13,14].

Among them, iron (II) selenide, which has been widely studied for years to unveil the high-temperature superconductivity in iron-based superconductors, is drawing increasing attention in electrochemistry because of (i) the high abundance of iron in the earth and (ii) its far higher electrical conductivity compared to oxides. Indeed, since Se atoms are far less electronegative than oxygen atoms, the valence band of selenides lies at a higher energy, resulting in a smaller band gap (less than 1 eV) than those of oxides including the same transition metal [15,16].

In this study, small particles of iron (II) selenide with rough surfaces, prepared using an unconventional, simple, easily reproducible, and cost-effective procedure, were tested for the first time for their supercapacitor properties [17]. In particular, a simple, one-step, solvothermal method was proposed to synthesize FeSe. After a broad characterization of the material, the electrochemical performance was investigated using cyclic voltammetry, galvanostatic charge/discharge tests, and electrochemical impedance spectroscopy carried out in a H_2_SO_4_ aqueous solution. The results of the electrochemical measurements enabled us to understand that the novel material exhibits outstanding supercapacitor performance and superior stability in the potential range from 0 to 1 V.

## 2. Materials and Methods

### 2.1. FeSe Preparation

To synthesize the material, 0.1 mmol 2,4-iron pentanedione (C_10_H_16_FeO_4_) and 0.1 mmol sodium selenide (Na_2_Se) were added to a Teflon autoclave. After adding the metal precursors, the preparation of the mixture was completed by adding 6.0 mL of water, 0.5 mL of cyclohexane (C_6_H_12_), 2.5 mL of dimethylformamide (C_3_H_7_NO), and 3.0 mL of ethylene glycol (C_2_H_6_O_2_) and then stirring the as-prepared mixture for an hour. The solvothermal process was carried out by maintaining the homogeneous mixture in an oven for 12 h at 180 °C. Afterward, black solid products were formed and then washed three times with deionized water and ethanol and, eventually, dried at 80 °C for 8 h.

### 2.2. Characterization

Scanning electron microscopy (SEM) was performed using a TESCAN-VEGA LMH (230 V) microscope equipped with an EDX probe. The X-ray powder pattern of the sample was obtained using a Bruker D8 X-ray diffractometer with monochromatic CuKa radiation. 

Cyclic voltammetry, galvanostatic charge/discharge tests, and electrochemical impedance spectroscopy, carried out in the frequency range of 10 kHz–0.1 Hz at a DC voltage of 0.6 V with an AC bias of 5 mV, were performed using an Autolab PGSTAT302N potentiostat in a three-electrode-setup-based electrolytic cell filled with a 1 M H_2_SO_4_ solution [18,19]. Saturated calomel, graphite, and a loadable glassy carbon ring (3 mm diameter) were adopted, respectively, as the reference, counter, and working electrodes [20]. The mass capacitance (Csp, F/g) of the electrode, a capacitance calculated in a three-electrode setup, which can be expected to differ significantly from the values exhibited in a two-electrode packaged cell [21], was evaluated using GCD curves according to the following equation [22]:

(1) $$Csp=\frac{i\cdot \Delta t}{m\cdot \Delta V}$$

where *i* is the discharge current, Δt is the discharge time, ΔV is the potential window, and *m* is the loading mass of the electrode material.

Moreover, the energy density (*E*, Wh/kg) and power density (*P*, W/kg) were calculated as follows:

(2) $$E=\frac{1}{2}Csp\cdot \;\Delta V^{2}\frac{1000}{3600\;}$$

(3) $$P=\frac{E}{\Delta t}\;$$

Before the tests, 4 mg of the synthesized material was mixed into 80 μL of a 5 wt% Nafion solution to prepare a stable and conductive homogenous ink to be partially loaded onto the glassy carbon electrode [6,23,24].

## 3. Results and Discussion

### 3.1. Morphological Characterization

**Figure 1:**
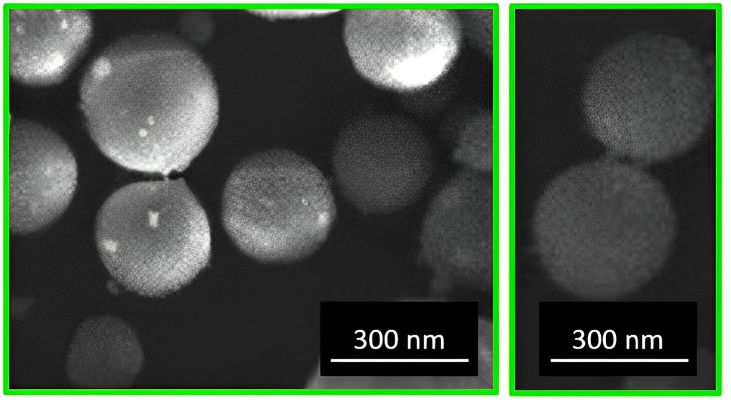
SEM images of FeSe.

The morphological and structural features of the FeSe sample were determined using a scanning electron microscope equipped with an EDX probe. In particular, the images in Figure 1 highlighted the formation of very small spherical particles with rough surfaces and an average diameter in the range of 170–300 nm.

**Figure 2:**
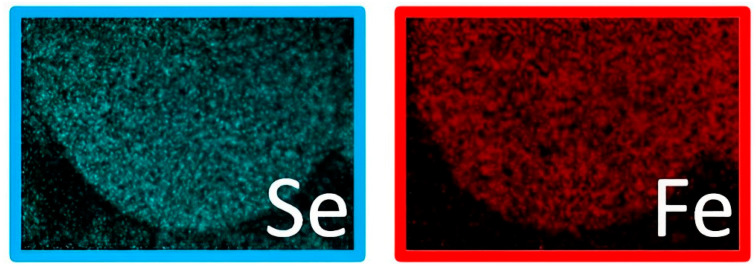
EDX maps of the Fe and Se elements of a single particle.

The corresponding EDX maps provided information on the elemental composition of the particles. In detail, Figure 2 shows a homogeneous distribution of Fe and Se elements within the sample, suggesting uniform electrochemical behavior.

**Figure 3:**
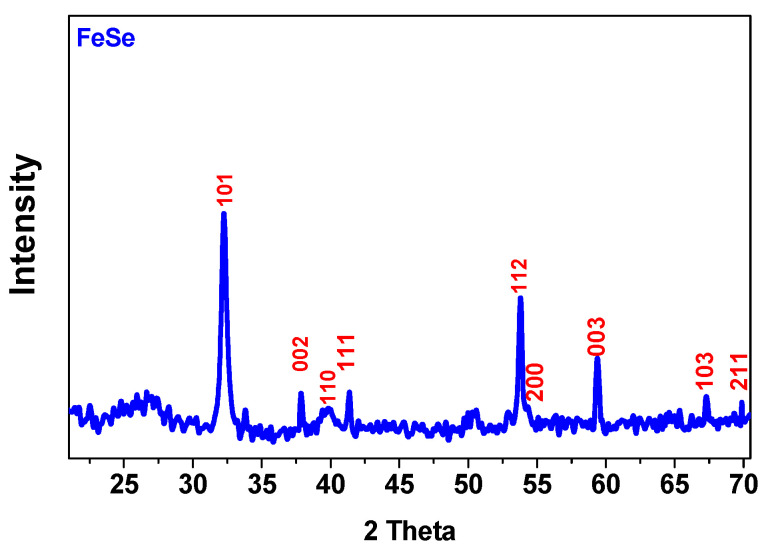
X-ray diffraction pattern of FeSe.

The X-ray diffraction pattern in Figure 3, showing the typical peaks of iron (II) selenide, confirmed the nature of the sample [25].

### 3.2. Electrochemical Characterization

**Figure 4:**
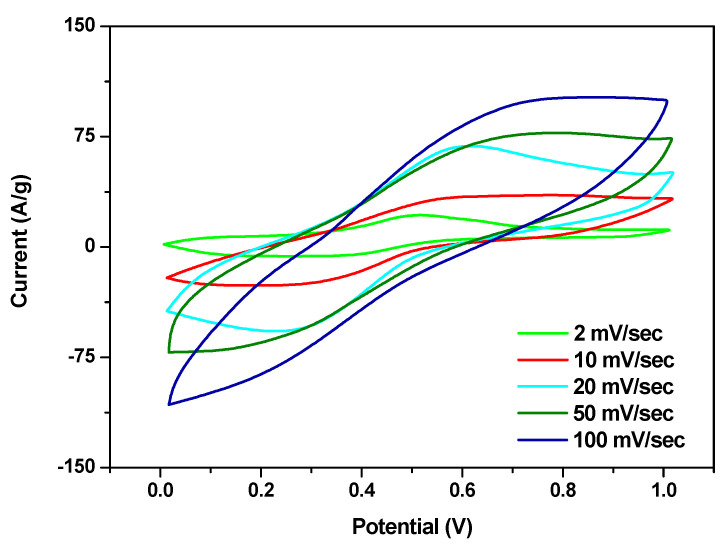
Cyclic voltammetry of FeSe at 2, 10, 20, 50, and 100 mV/s in the range of 0–1 V.

Cycling voltammetry and galvanostatic charge/discharge tests were performed to evaluate the electrochemical properties of the synthesized sample. In Figure 4, the CV curves of FeSe, recorded at different scan rates in a 0.5 M H_2_SO_4_ aqueous electrolyte solution, are reported. These curves recorded in the 0–1 V window, although not rectangular, show a near mirror-image current response without highly visible peaks due to the occurrence of surface redox reactions, suggesting good supercapacitor behavior.

**Figure 5:**
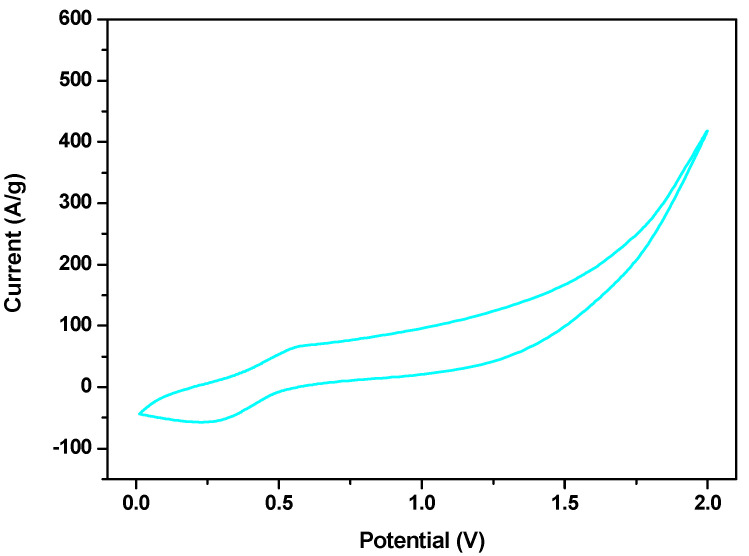
Cyclic voltammetry of FeSe at 20 mV/s in the range of 0–2 V.

CV curves for FeSe were also recorded in a wider voltage range (0–2 V), at a scan rate of 50 mV/sec (see Figure 5), proving that the sample still maintained its supercapacitor behavior over 1 V.

**Figure 6:**
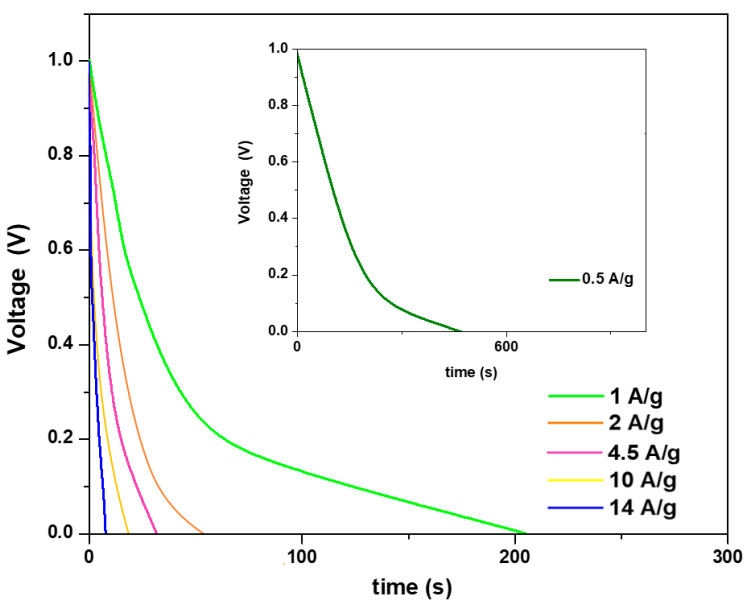
Discharge curves of FeSe at different current densities.

The specific capacitance was evaluated from the galvanostatic discharge curves (see Figure 6) by means of Equation (1). In particular, from the discharge curve, at a current density of 0.5 A/g, a specific capacitance of 280 F/g was obtained. The specific capacitance, as expected, decreased at increasing current densities to 185 F/g at 1 A/g and to 72 F/g at 14 A/g. Indeed, the higher the current density, the shorter the time for the electrolyte ions to diffuse into the electrode channels and, therefore, the smaller the portion of the active material’s surface area they can access. This results in decreased capacitances. In detail, a reduction in the capacitance of about 30% was obtained by doubling the charge/discharge current density.

**Figure 7:**
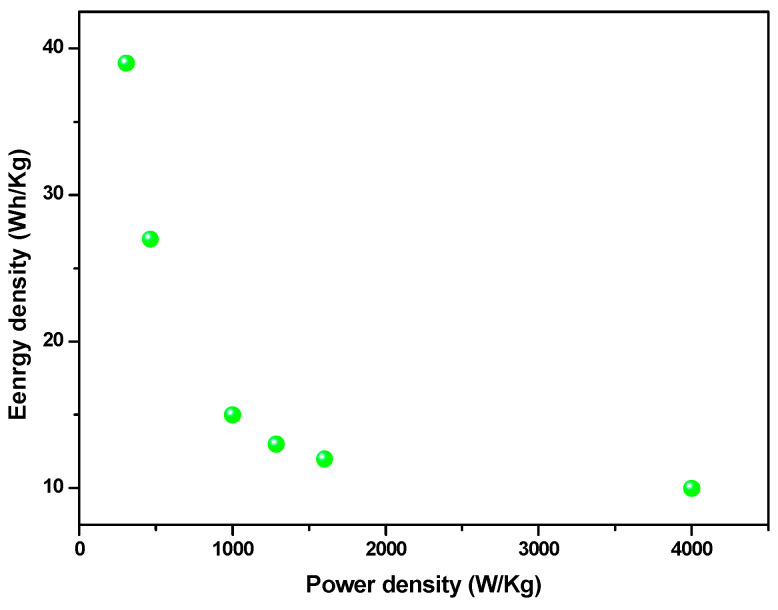
Ragone plot of FeSe as current density varies in the range of 0.5–18 A/g.

**Figure 8:**
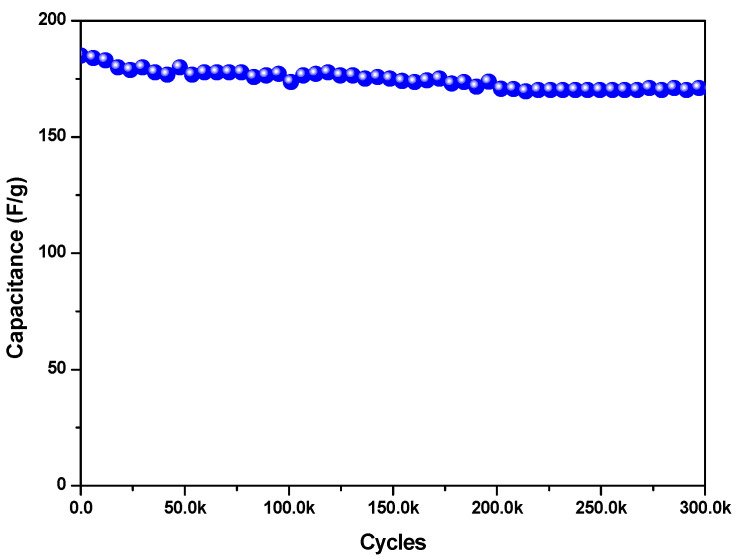
Capacitance vs. number of cycles of FeSe at 1 A/g.

The energy and power densities were also calculated using Equations (2) and (3) and are represented in the Ragone plot in Figure 7. The material showed an energy density of 48 Wh/kg, which decreased to 17 Wh/kg as the galvanostatic charge/discharge current density increased from 0.5 A/g to 18 A/g, while the power density increased from 470 W/Kg to 7300 W/Kg in the same current density range, confirming the excellent performance of the low-cost and environmentally friendly FeSe small-particle material. Furthermore, the electrode retained about 92% of its initial capacitance after 30,000 cycles (Figure 8), indicating a long cycle life with rather high stability.

In Table 1, the supercapacitor performance of the synthesized sample as well as those of some of the best-performing Se-based materials in the literature are reported. It appears that the material reported in this study ranks among the best ones, even in comparison with finer materials and materials produced with less-scalable processes. The produced sample shows a rather high specific capacitance at a low current density and, above all, one of the most significant durabilities in terms of capacitance retention. This is likely due to the strong Fe-Se covalent bonding and the electrode’s low internal resistance (see the electrochemical impedance spectroscopy results reported below), which increases the stability of the material over time [26].

**Figure 9:**
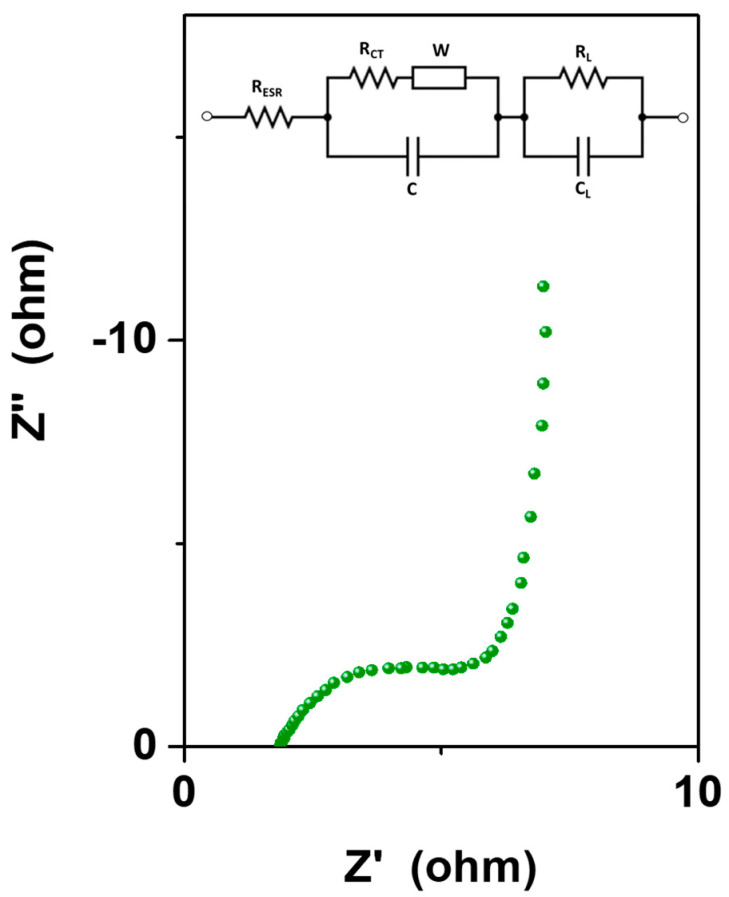
Nyquist plot of FeSe at a DC voltage of 0.6 V with an AC bias of ±0.005 V. In the inset, the electrical equivalent circuit. R_ESR_ = equivalent series resistance, R_CT_= interfacial charge transfer resistance, C = electrode capacitance, W = Warburg impedance, R_L_= low-frequency leakage resistance, C_L_ = low-frequency capacitance.

Furthermore, electrochemical impedance spectroscopy was performed to determine the resistivity as well as the charge-transfer and ion diffusion behavior of the prepared material. Figure 9 reports the Nyquist plot of the sample, with the corresponding electrical equivalent circuit, showing an inclined line in the low-frequency region and a quasi-semicircle in the high-frequency region. The arc describes the charge transfer resistance at the electrode/electrolyte interface (*R*_CT_) and the interfacial capacitance (*C*) connected in parallel at the electrode/electrolyte interface, while the inclined portion of the curve (about 45°) in the lower-frequency region is ascribed to the Warburg impedance (W), which refers to the resistance to the ion diffusion/transport from the electrolyte to the electrode surface [36]. At very low frequencies, an ideally polarizable capacitance (C_L_) would give rise to a straight line parallel to the y axis. However, in this case, the curve is slightly inclined, suggesting the presence of a low-frequency leakage resistance (R_L_) in parallel with the capacitance. In the high-frequency region, the intercept on the Z′ axis represents the equivalent series resistance, which refers to the combination of the internal resistance of the electrode and the ohmic resistance of the electrolyte as well as the contact resistance between the current collector and the electrode. The sample exhibited an R_ESR_ equal to ~2 Ω, indicative of a rather low internal resistance.

## 4. Conclusions

In summary, in this study, a simple, easily scalable and reproducible, and cost-effective synthetic procedure was adopted for the production of an FeSe electrode of controlled size and morphology for supercapacitor applications. In particular, the reported procedure enabled the synthesis of spherical particles with rough surfaces with an average diameter in the range of 170–300 nm. As a result of the electrochemical tests, a very high specific capacitance of 280 F/g and a rather high energy density of 48 Wh/kg at 0.5 A/g were obtained, along with a maximum power density of 7300 W/Kg and an extremely high durability, even when compared with finer materials and materials produced with less-scalable processes. The reported excellent performance of FeSe can be attributed to a combination of factors, including the superior electrical conductivity of the material, the very high surface area, and the increased porosity due to the reduced size of the rough particles, as well as the strong Fe-Se interaction and the low internal resistance of the material. In conclusion, the encouraging results obtained with the material prepared in this study, as well as those reached in some of the currently published studies, suggest the possibility of exploring transition metal selenide-based materials as valid and effective alternatives for supercapacitor applications. 

## Figures and Tables

**Table 1:** Supercapacitor performances of the FeSe sample and some of the best-performing transition metal selenide-based materials in the literature.

Se-Based Material (with Average Size)	CV Potential Window (V)	GCD Current Density (A/g)	Specific Capacitance (F/g_sample_)	Energy Density (Wh/kg)	Power Density (W/kg)	Capacitance Retention	Ref.
**SnSe_2_ nanodisks (8 nm)**	0 ÷ 0.45 vs. SCE	0.5	168	-	-	retains about 99.0% after 1000 cycles at 1 A/g	[27]
**SnSe_2_ nanosheets (2 nm)**	0 ÷ 0.6 vs. SCE	0.5	228	-	-	retains about 99.2% after 1000 cycles at 1 A/g	[27]
**NiSe nanoparticles (23 nm)**	−0.2 ÷ 0.4 vs. SCE	1	705	-	-	retains about 92% after 2500 cycles at 5 A/g	[28]
**MoSe_2_ nanoflakes /Mo Se_2_ nanorods (500 nm)**	0 ÷ 0.6 vs. Hg/HgO	2	133	36.2 (MoSe_2_ NRs ∥ MoSe_2_ NFs device)	1400 (MoSe_2_ NRs ∥ MoSe_2_ NFs device)	retains about 92% after 2000 cycles at 2 A/g	[29]
**GeS_2_ nanobelts (125 nm)**	0.1 ÷ 0.65 vs. Hg/HgO	1	300	-	-	retains about 99.3% after 2000 cycles at 1 A/g	[30]
**CuSe nanosheets (15 nm)**	−0.4 ÷ 0.2 vs. SCE	0.2	209	-	-	retains about 90% after 10,000 cycles at 1 A/g	[31]
**CdSe microspheres (300 nm)**	0 ÷ 0.8 vs. SCE	0.2 mA	1.285 mFcm^−2^	4.015	-	retains about 83.7% after 2000 cycles	[32]
**MnSe nanoparticles (25 nm)**	0 ÷ 0.8 vs. Ag/AgCl	0.1 mA/cm^−2^	96.76	8.60	47.05	Specific capacitance increases slightly (103.40%) after 2000 cycles	[33]
**CeSe_2_ nanopebbles/MWCNTs (23.7 nm)**	−0.9 ÷ 0 vs. Ag/AgCl	2 mA/cm^−2^	451.4	36.3 (CeSe_2_/MWCNTs ∥ CeSe_2_/MWCNTs device)	2800 (CeSe_2_/MWCNTs ∥ CeSe_2_/MWCNTs device)	retains about 70.7% after 4000 cycles at 100 mV/s	[34]
**RuSe_2_@550 nanoparticles (7 nm)**	0 ÷ 0.7 vs. SCE	0.5	786.4	44.8 (RuSe_2_@500 ∥ carbon powder device)	400 (RuSe_2_@500 ∥ carbon powder device)	retains about 78.2% after 10,000 cycles	[35]
**very small FeSe particles (235 nm)**	0 ÷ 1 vs. SCE	0.5	280	39	306	retains about 92% after 30,000 cycles at 1 A/g	This work

## Data Availability

Not applicable.
